# Silver nanoparticles decorated ZnO–CuO core–shell nanowire arrays with low water adhesion and high antibacterial activity

**DOI:** 10.1038/s41598-023-37953-w

**Published:** 2023-07-03

**Authors:** Andreea Costas, Nicoleta Preda, Irina Zgura, Andrei Kuncser, Nicoleta Apostol, Carmen Curutiu, Ionut Enculescu

**Affiliations:** 1 National Institute of Materials Physics, Atomistilor 405A, 077125 Magurele, Romania; 2 Microbiology Immunology Department, Faculty of Biology, University of Bucharest, Aleea Portocalelor 1-3, 060101 Bucharest, Romania

**Keywords:** Nanowires, Semiconductors

## Abstract

Nanostructured surfaces based on silver nanoparticles decorated ZnO–CuO core–shell nanowire arrays, which can assure protection against various environmental factors such as water and bacteria were developed by combining dry preparation techniques namely thermal oxidation in air, radio frequency (RF) magnetron sputtering and thermal vacuum evaporation. Thus, high-aspect-ratio ZnO nanowire arrays were grown directly on zinc foils by thermal oxidation in air. Further ZnO nanowires were coated with a CuO layer by RF magnetron sputtering, the obtained ZnO–CuO core–shell nanowires being decorated with Ag nanoparticles by thermal vacuum evaporation. The prepared samples were comprehensively assessed from morphological, compositional, structural, optical, surface chemistry, wetting and antibacterial activity point of view. The wettability studies show that native Zn foil and ZnO nanowire arrays grown on it are featured by a high water droplet adhesion while ZnO–CuO core–shell nanowire arrays (before and after decoration with Ag nanoparticles) reveal a low water droplet adhesion. The antibacterial tests carried on *Escherichia coli* (a Gram-negative bacterium) and *Staphylococcus aureus* (a Gram-positive bacterium) emphasize that the nanostructured surfaces based on nanowire arrays present excellent antibacterial activity against both type of bacteria. This study proves that functional surfaces obtained by relatively simple and highly reproducible preparation techniques that can be easily scaled to large area are very attractive in the field of water repellent coatings with enhanced antibacterial function.

## Introduction

Semiconducting nanowires are one of the most outstanding one-dimensional nanomaterials being featured by a high surface to volume ratio and tunable physicochemical characteristics, which are making them very attractive for a wide range of applications including (opto-) electronics, photovoltaic cells, (photo-) catalysis, (bio-) sensing, (bio-) medicine, surfaces with controlled wettability, and so on^1,2^.

Metal oxides are highly competitive materials owed to their interesting features like abundance, low environmental impact and rich family of morphologies with controlled sizes that can be synthesized by various wet and dry aproaches^3–8^. Hence, metal oxide nanowires based on ZnO or/and CuO can be engineered by combining different preparation methods^7–21^ such as thermal oxidation in air, radio frequency (RF) magnetron sputtering, plasma assisted thermal vapor deposition, ultrasonic spray-assisted chemical vapor deposition, chemical spray pyrolysis, chemical synthesis, hydrothermal, etc. to yield attractive functional nanomaterials, which can be applied in field effect transistors^7,9,10^, diodes^10^, photodetectors^11–14^, solar cell^15^, photocatalysis^16^, surfaces with special wetting properties^17,19–21^, etc. Furthermore, multicomponent nanowires with complex architectures developed by decoration of metal oxide nanowires with metal nanoparticles is an effective route for expanding and enhancing the nanowires functionalities^22–26^.

Metal oxide nanowire arrays based on ZnO and CuO can be easily grown by thermal oxidation in air directly on zinc foils^9,14,16,17^ and copper foils^8,10,13,16^, respectively. Thermal oxidation in air is a straightforward and environmental friendly preparation path, which can be adapted for large-scale production of metal oxide nanowires. This technique can be also regarded as a viable solution to micro/nanostructuring a metallic surface, specific properties and new functionalities, different from those presented by the pristine metal bulk material, being achieved by the fabricated micro/nanostructured surfaces. In the recent years, many researches have been focused on the development of such micro/nanostructured surfaces for bactericidal applications^27–29^. It is known that ZnO and CuO present antibacterial activity separately as single components^30^ or combined as nanocomposites^31–33^, several studies emphasizing the potential application of ZnO and CuO nanowires in the antibacterial area^34–37^. ZnO is a n-type compound with a wide band gap (~ 3.37 eV)^5^ while CuO is a p-type compound with a narrow band gap (~ 1.2 eV)^6^, the p–n junction obtained by joining these two semiconductors improving the separation of the charge carrier pairs. Thus, in a previous study we reported on the fabrication of staggered gap radial heterojunctions based on ZnO–Cu_x_O core–shell nanowires by covering thermally oxidized ZnO nanowires with an optimum thickness of Cu_x_O layer by RF magnetron sputtering, these nanomaterials finding applications as diodes^14^ or as water stable photocatalysts^16^. Although, the addition of Ag to composites based on ZnO and CuO can improve the separation of the electron–hole pairs, only few papers were reported on the nanocomposites such as Ag-ZnO^38^, Ag-CuO^38^, Ag and CuO impregnated on Fe doped ZnO^39^, Cu_2_O and Ag co-modified ZnO^40^ with an enhanced antibacterial activity, these nanomaterials being synthesized by wet chemical methods. As regards the preparation of one-dimensional multicomponent nanostructures (wires, rods, tubes, etc.) based on ZnO, CuO and Ag, despite the fact that the wet chemistry consume and discharge different chemicals with potentially adverse effects on the environment, many studies^41–47^ were carried on their synthesis by wet chemical paths (co-precipitation, chemical bath deposition electrochemical deposition, hydrothermal, sol–gel etc.) and on their applications in superhydrophobic coatings, photocatalysis, dye photodegradation, trace pesticide detections, etc. In comparison to the wet chemistry, the development of metal oxide nanowires by dry techniques such as thermal oxidation in air, radio frequency (RF) magnetron sputtering and thermal vacuum evaporation can be considered “clean” environmental friendly routes being solution-free preparation pathways, which do not involve hazardous raw materials and liquid solvent and do not produce harmful by-products.

Usually, the performance of the antibacterial surfaces depends on parameters such as surface morphology, surface chemical composition and surface wettability, these being mainly responsible for the most common bacteria-killing phenomena: contact-killing surfaces, nanoprotrusions and superhydrophobic surfaces^29^. For example, the water droplets can easily roll-off on superhydrophobic surfaces with water angle greater than 150° carrying away the bacteria, the nanoprotrusions can mechanically damage the bacteria cells while the use of materials well known for their pronounced antibacterial activity (metals, metal oxides) can lead to a chemical contact killing of bacteria. A priori, due to the synergy effect between the individual bacteria-killing phenomena, a hydrophobic nanostructured metal surface containing sharp nanostructures like metal oxide nanowires decorated with metal nanoparticles can be an ideal candidate for inexpensive coatings with enhanced antibacterial function. Additionally, the formation of different type of junctions between the components can also favors the separation of charge carrier pairs, augmenting the generation of highly reactive oxygen (ROS), generally these species playing the central role in the mechanisms responsible for the bacteria killing.

In this context, the present study is focused on the development of nanostructured surfaces based on silver nanoparticles decorated ZnO–CuO core–shell nanowire arrays by combining thermal oxidation in air, RF magnetron sputtering and thermal vacuum evaporation. To our knowledge, no attempt was made for developing multicomponent nanowires based on ZnO, CuO and Ag by combining these three dry techniques that are frequently used in the preparation of inorganic nanostructures. Furthermore, this preparation approach presents two key advantages: (i) can be applicable to large-area for obtaining multicomponent nanowire arrays with high density and good uniformity and (ii) can be regarded as a viable strategy for tuning the architecture and composition of the multicomponent nanowires in order to achieve desired properties and improved performances. Moreover, in comparison to other preparation methods of ZnO nanowires that use chemical reagents and require additional steps for the deposition of the precursor layers (metallic zinc films or metal oxide seed layers for promoting the growth of the nanowire structures)^18–21^, the thermal oxidation of zinc foils offers the main advantage of growing ZnO nanowires directly on them, these metal oxide functionalized metallic substrates being attractive for applications where properties such as anticorrosion, self-cleaning, hydrophobicity or antibacterial are demanded. Hence, ZnO nanowire arrays were grown directly on zinc foils by thermal oxidation in air, further these were coated with a CuO layer by RF magnetron sputtering, the obtained ZnO–CuO core–shell nanowires being decorated with Ag nanoparticles by thermal vacuum evaporation. The morphological, compositional, structural, optical, surface chemistry, wetting and antibacterial activity properties of the prepared samples were evaluated. The nanostructured surfaces based on nanowire arrays present exceptional antibacterial activity against both *Escherichia coli* (a Gram-negative bacterium) and *Staphylococcus aureus* (a Gram-positive bacterium). Most probably, the synergetic effect between the sharp morphology of the nanowires and the low water droplet adhesion behavior of the nanostructured surfaces is responsible for the excellent antibacterial effect.

## Experimental section

### Preparation of Ag nanoparticles-decorated ZnO–CuO core–shell nanowire arrays

**Figure 1:**
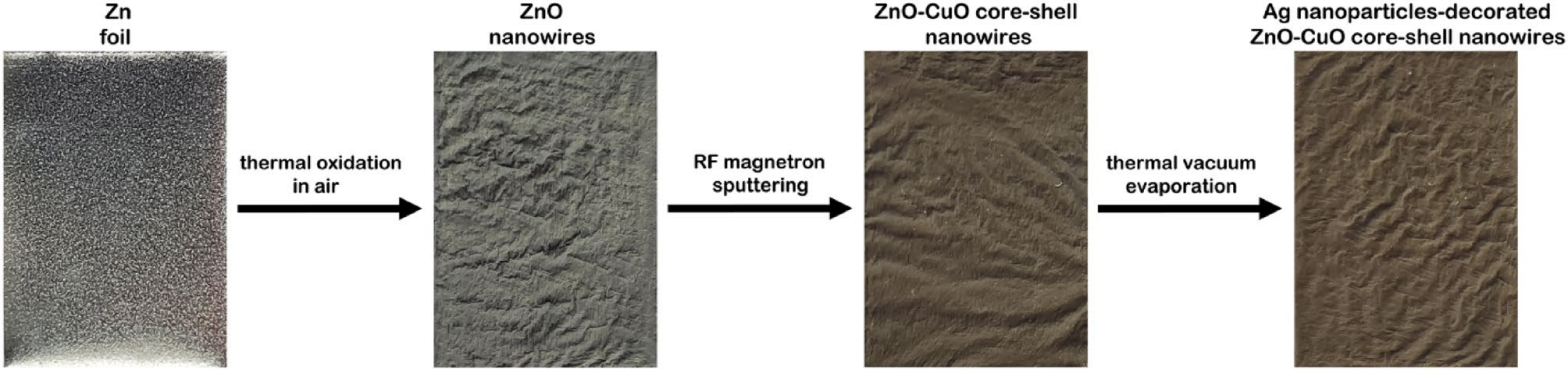
Schematic representation of the steps involved in the functionalization of the zinc foil with nanowires by dry techniques and the photographs of the zinc foil before and after each applied functionalization process.

Zinc foil (Alfa Aesar, 99.98% purity) was cut into pieces of approximately 2 × 1.5 cm^2^, subsequently these were cleaned in acetone and isopropyl alcohol for 5 min in an ultrasonic bath, washed in deionized water and dried under nitrogen gas flow. ZnO nanowires were prepared by thermal oxidation in air of Zn foil samples at 500 °C for 12 h. Then, the ZnO nanowires were coated with a CuO layer by RF magnetron sputtering using a copper oxide sputtering target (Kurt J. Lesker Company Ltd., 99.7% purity). Before each deposition, the metal oxide target was pre-sputtered for 30 min in order to remove any possible contamination from its surface. During the deposition, the power applied on the magnetron was 100 W and the pressure in the chamber was 5.4 × 10^−3^ mbar in an Ar atmosphere with a purity of 9.6 (99.9999%) from Linde. Further, the ZnO–CuO core–shell nanowires were decorated with Ag nanoparticles by thermal vacuum evaporation using a silver wire (Aldrich, 99.9% purity), the pressure in the chamber being 3 × 10^−6^ mbar. The photographs of the investigated samples are presented in Fig. 1, the visual/color detail being specific for each preparation step. Thus, the Zn foil lost the metallic luster acquiring the typical white color of ZnO after the thermal oxidation, dark brown color after the coating with CuO layer and light brown color after the decoration with Ag nanoparticles. Also, it can be noticed that the flat aspect of the foil is changed into a wrinkled one after the thermal oxidation process.

### Characterization techniques

The prepared samples were characterized by complementary techniques. The surface morphology and its elemental composition were studied using a Zeiss Merlin Compact field emission scanning electron microscope (FESEM) and a Zeiss EVO 50XVP scanning electron microscope equipped with an energy dispersive X-ray analysis (EDX) QUANTAX Bruker 200 accessory for composition. Furthermore, the morphology, atomic structure and local chemical composition structure at nanoscale level were analyzed by transmission electron microscopy (TEM), EDX including elemental mapping in scanning transmission electron microscopy (STEM) and selected area electron diffraction (SAED) using a Cs probe-corrected JEM ARM 200F analytical electron microscope. The crystalline phase was investigated using a Bruker AXS D8 Advance X-ray diffractometer (XRD) with Cu Kα radiation (λ = 0.154 nm), the source being operated at 40 kV and 40 mA. The reflectance and photoluminescence (PL) were evaluated using a Perkin-Elmer Lambda 45 UV–VIS spectrophotometer equipped with an integrating sphere for reflectance and a FL 920 Edinburgh Instruments spectrometer with a 450 W Xe lamp excitation and double monochromators on both excitation and emission for photoluminescence (λ_exc_ = 350 nm), respectively. The surface chemistry was analyzed by X-Ray photoelectron spectroscopy (XPS) with an AXIS Ultra DLD (Kratos Surface Analysis) setup using Al K_α1_ (1486.74 eV) radiation produced by a monochromatized X-Ray source at operating power of 225 W (15 kV × 15 mA). In the analysis chamber, the base pressure was at least 1.0 × 10^–8^ mbar. A flood gun operating at 1.5 A filament current, 2.7 V charge balance and 1.0 V filament bias was utilized for achieving the charge compensation. Hybrid lens mode, 40 eV pass energy and a slot aperture were applied for recording high-resolution core level spectra, Voigt profiles (singlets or doublets) being used in their deconvolution according to previously described methods^48^.

The wetting properties were assessed by measuring the static contact angle (CA) with a Drop Shape Analysis System, model DSA100 from Kruss GmbH. The surface free energy (SFE) with its polar (γ^p^) and dispersive (γ^d^) components for the solid surface was evaluated using the apparent CA values measured between a test liquid and the sample surface. Water and diiodomethane were used as test liquids for assuring an accurate evaluation of the polar and dispersive components of the surface free energy of the investigated samples. Thus, a droplet from the test liquid was placed on the sample surface via a blunt-end, stainless steel needle (outer diameter of 0.5 mm) attached to a syringe pump controlled by the DSA3^®^ software supplied with the instrument. The volume of the droplet was 1 μL for diiodmethane (DIM) while that for water (W) was increased from 1 to 5 μL (in some cases, the water droplet did not stick to the surface in order to measure the CA so the drop volume was gradually increased until this adheres to the surface). CA was measured by fitting a polynomial equation of second degree or a circle equation to the shape of the sessile drop and then calculating the slope of the tangent to the drop at the liquid–solid vapor interface line. The detailed processes of the behavior of the water droplet adhesion at the sample surface was recorded by a video camera tilted at 2–3° with respect to the plane of the sample surface supporting the droplet. Hence, the surface free energy was estimated based on the Owens–Wendt relationship *γ*_*SL*_ = *γ*_*L*_ + *γ*_*S*_*–2(γ*_*L*_^*d*^*γ*_*S*_^*d*^*)*^*1/2*^*–2(γ*_*L*_^*p*^*γ*_*S*_^*p*^*)*^*1/2*^, where *γ*_*SL*_ is the interfacial energy between solid and liquid, *γ*_*L*_ and *γ*_*S*_ are the surface energy of liquid and solid, respectively, *γ*_*L*_^*p*^ and *γ*_*S*_^*p*^ are the polar component of surface energy of liquid and solid, respectively and *γ*_*L*_^*d*^ and *γ*_*S*_^*d*^ are the dispersive component of surface energy of liquid and solid, respectively^49^. The *γ*_*L*_^*p*^, *γ*_*L*_^*d*^* γ*_*L*_ values^50^ for the test liquids are: *γ*_*water*_^*p*^ = 51 mN/m, *γ*_*water*_^*d*^ = 21.8 mN/m and *γ*_*water*_ = 72.8 mN/m and *γ*_*diiodomethane*_^*p*^ = 2.3 mN/m, *γ*_*diiodomethane*_^*d*^ = 48.5 mN/m and *γ*_*diiodomethane*_ = 50.8 mN/m. The adhesion work (*W*_*ad*_) between the liquid droplet and the sample surface was estimated based on Young–Dupre relationship *W*_*ad*_ = *γ*_*L*_*(1* + *cosθ)*, where θ is the CA of liquid on investigated solid surface^51,52^. The measurements were performed in duplicate at room temperature, the CA mean value being used for evaluating the surface free energy and the adhesion work. Roll-off angles were measured with a goniometer in order to control the tilt angle, the orthoscopic images being taken with a commercial photocamera.

The antibacterial activity was evaluated against non-pathogenic *Escherichia coli* (*E. coli* ATCC 25922) strain (Gram-negative bacteria) and non-pathogenic *Staphylococcus aureus* (*S. aureus* ATCC 25923) strain (Gram-positive bacteria), both in planktonic state. Thus, 2 ml tryptic soy broth (TSB) and 20 μl microbial suspension with 0.5 McFarland density (1.5 × 10^8^ CFU/mL) were added on the sterilized investigated samples placed in 6-well plates. After the incubation (carried at 37 °C for 24 h), decimal dilutions obtained from the suspension recovered in sterile saline were seeded in triplicate (3 replicates of 10 µl each) on agar medium for quantifying the colony forming units (CFU)/ml. It has to be noted that each replicate was performed in triplicate, the experiments being repeated 2 times in triplicates. The antibacterial efficiency of the investigated samples was calculated in terms of percentage of bacterial cell reduction (R, %) determined as follows: *R %* = *[(CFU*_*c*_*-CFU*_*p*_*)/CFU*_*c*_*]* × *100*; where *CFU*_*c*_ and *CFU*_*p*_ represent the numbers of CFU/ml for the control and each investigated sample.

## Results and discussion

The surface morphology of the metallic substrate after each functionalization step was analyzed by FESEM, the images being presented at different magnifications (Figs. S1 and 2). At lower magnification (area having width × height of ~ 250 × 160 µm), the FESEM images (Fig.  S1) reveal that after each step, the zinc foil is uniformly covered by nanowires.

**Figure 2:**
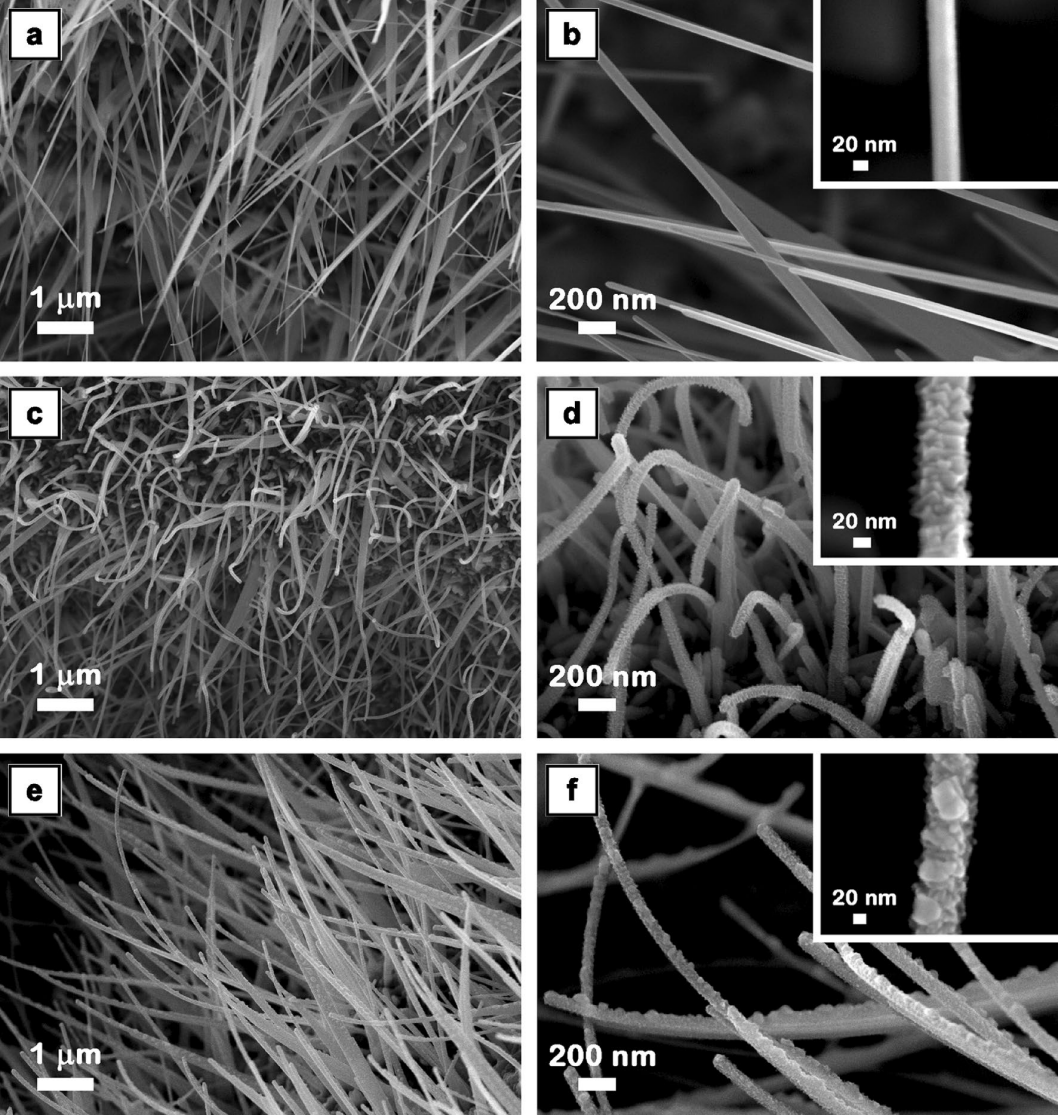
FESEM images at two magnifications of ZnO nanowires (**a**,**b**), ZnO–CuO core–shell nanowires (**c**,**d**) and Ag nanoparticles-decorated ZnO–CuO core–shell nanowires (**e**,**f**). Insets: FESEM images at higher magnification of a single nanowire from the corresponding samples.

At higher magnification, the FESEM images (Fig. 2) indicate that the deposition parameters employed in the RF magnetron sputtering and thermal vacuum evaporation were adequately chosen in order to preserve the typical cylindrical form and the high aspect ratio of the nanowires during each preparation step. Thus, after thermal oxidation in air, the ZnO nanowires are densely grown on the Zn foil surface having the diameters down to 30 nm and the lengths varying from several micrometers to tens of micrometers. Further, the deposition of a granular nanostructured uniform CuO layer on the surface of the ZnO nanowires by RF magnetron sputtering results in an increase of their diameters from ~ 30 nm to ~ 60 nm, the thickness of the CuO shell being estimated at ~ 15 nm. Then, the decoration of ZnO–CuO core–shell nanowires with Ag nanoparticles by thermal vacuum evaporation leads in the deposition of metallic aggregates with irregular shape and sizes of ~ 40 nm, the thickness of the nanowire segments containing ZnO core–CuO shell–Ag nanoparticles increasing up to ~ 70 nm.

**Figure 3:**
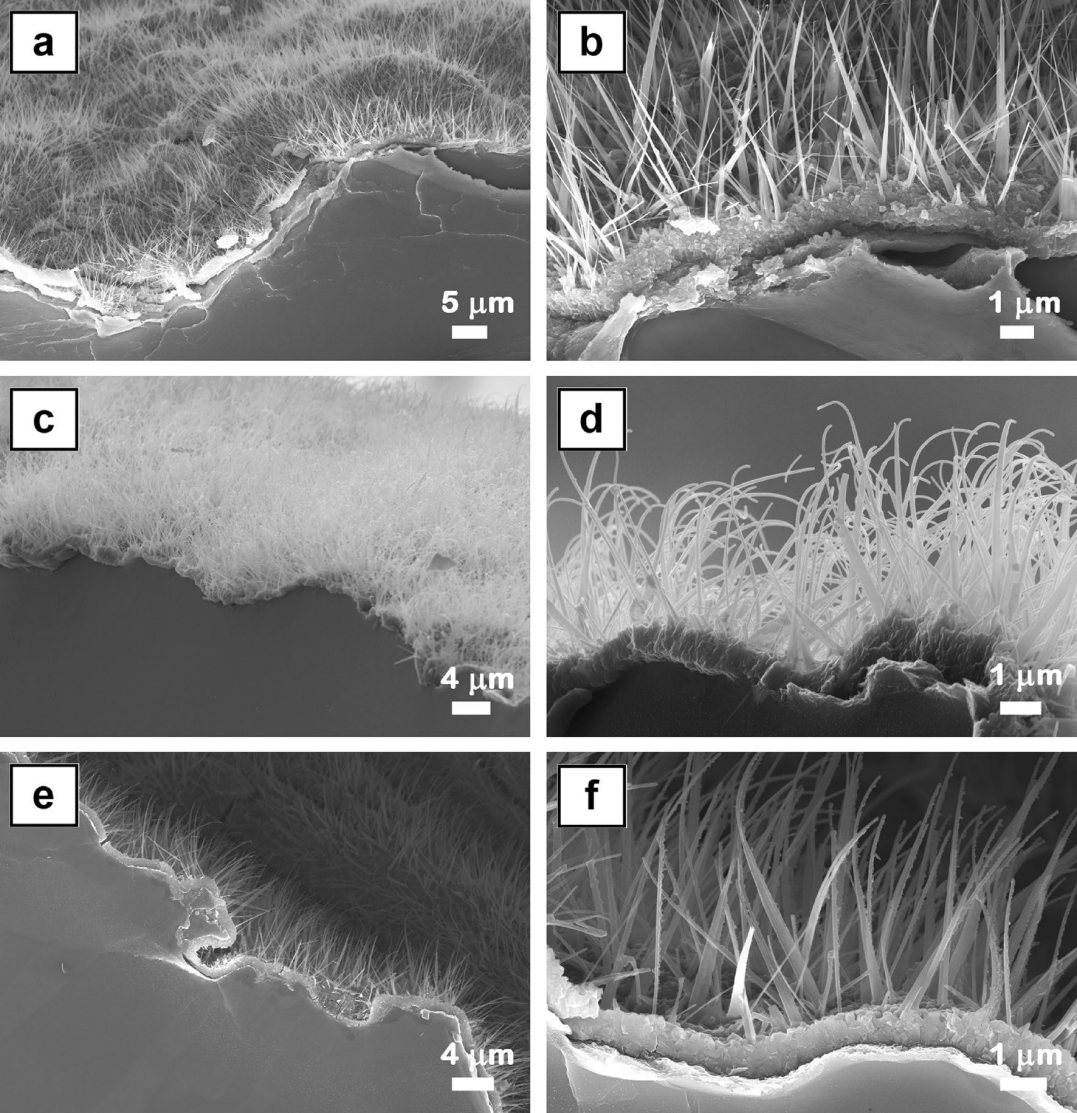
Cross-sectional FESEM images of ZnO nanowires (**a**,**b**), ZnO–CuO core–shell nanowires (**c**,**d**) and Ag nanoparticles-decorated ZnO–CuO core–shell nanowires (**e**,**f**).

The cross-sectional FESEM images (Fig. 3) emphasize the formation mechanism of the ZnO nanowires by thermal oxidation in air of zinc foil at 500 °C (temperature between the melting point of Zn (420 °C) and boiling points of Zn (907 °C)) based on a liquid–solid process in which the liquid Zn reacts with the oxygen molecules from air to form solid ZnO nanoclusters that grow via surface diffusion of Zn^53^. As can be seen in Fig. 3, the ZnO nanoclusters are assembled in a nanostructured ZnO film featured by a thickness of ~ 1 µm at the end of the thermal oxidation process. Hence, the formation mechanism of ZnO nanowires involves: (i) the formation of a thin layer of liquid Zn on the surface of the Zn substrate; (ii) the adsorption of the oxygen molecules on the surface of the liquid Zn in order to form solid ZnO nanoclusters, which further act as seeds in the growth of the ZnO nanowires; (iii) the surface diffusion of Zn ions along the side wall of the nanowire, their reaction with oxygen resulting to a simultaneous growth in both the axial and radial directions of the nanowires (the significant amount of Zn ions incorporated into the nanowire near the root region to the tip explaining why most of the nanowires become thinner at the tip).

**Figure 4:**
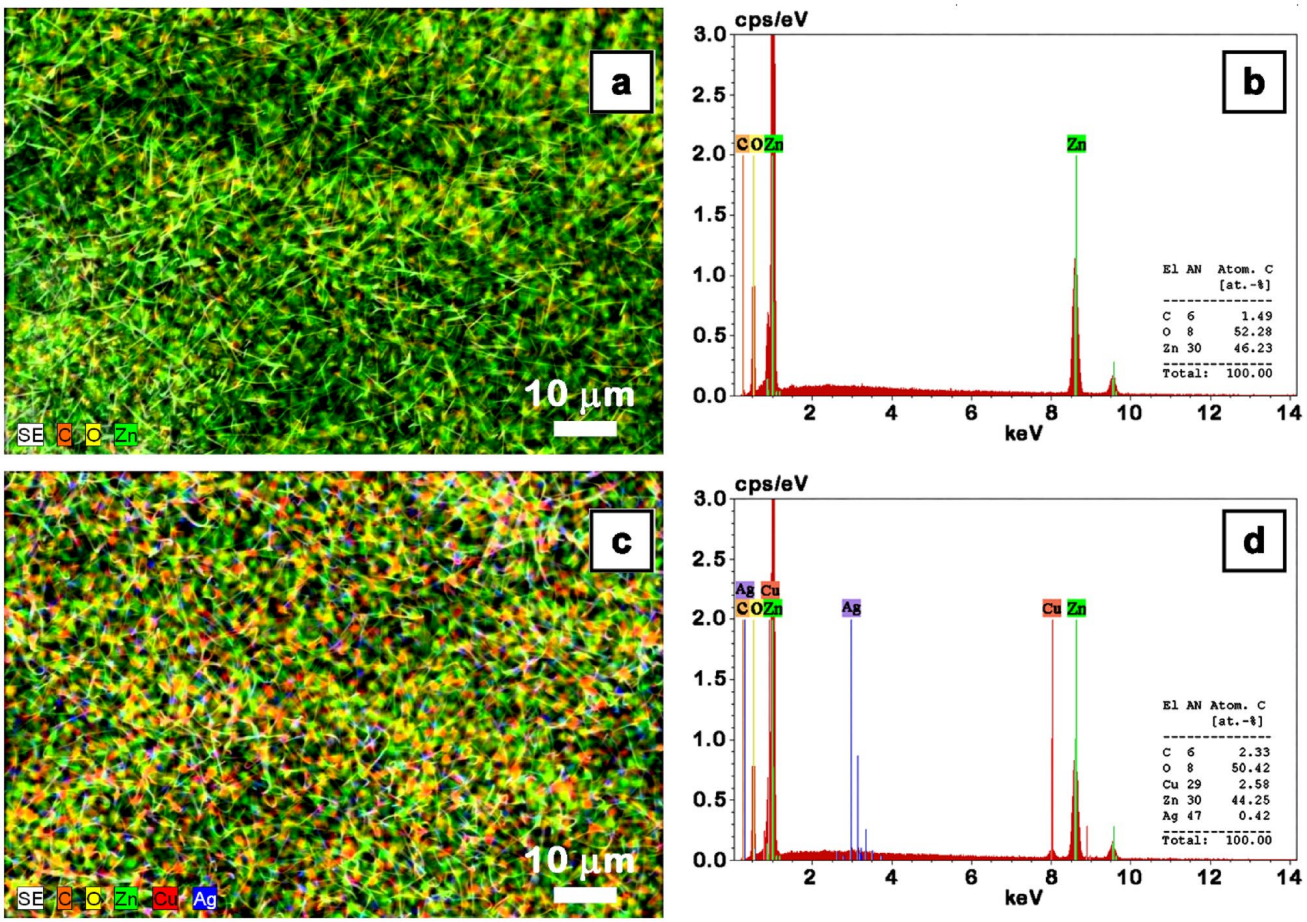
EDX mapping images (**a**,**c**) and EDX spectra (**b**,**d**) of the ZnO nanowires (**a**,**b**) and Ag nanoparticles-decorated ZnO–CuO core–shell nanowires (**c**,**d**).

The elemental distribution and the composition of the ZnO nanowire arrays and Ag nanoparticles decorated ZnO–CuO core–shell nanowire arrays samples were assessed by EDX spectroscopy, the EDX mapping images and the corresponding spectra being given in Fig. 4.

The EDX mapping images illustrate an uniform distribution of the chemical elements on the investigated surfaces. The EDX spectrum of ZnO nanowires reveals the presence of Zn and O elements while the EDX spectrum of Ag nanoparticles decorated ZnO–CuO core–shell nanowire arrays discloses the simultaneously presence of the signals associated to Zn, Cu, Ag and O elements. Based on the EDX analysis, the atomic percentage of the elements contained in the analyzed samples was estimated at ~ 44–46% for Zn, ~ 2.5% for Cu and ~ 0.4% for Ag.

**Figure 5:**
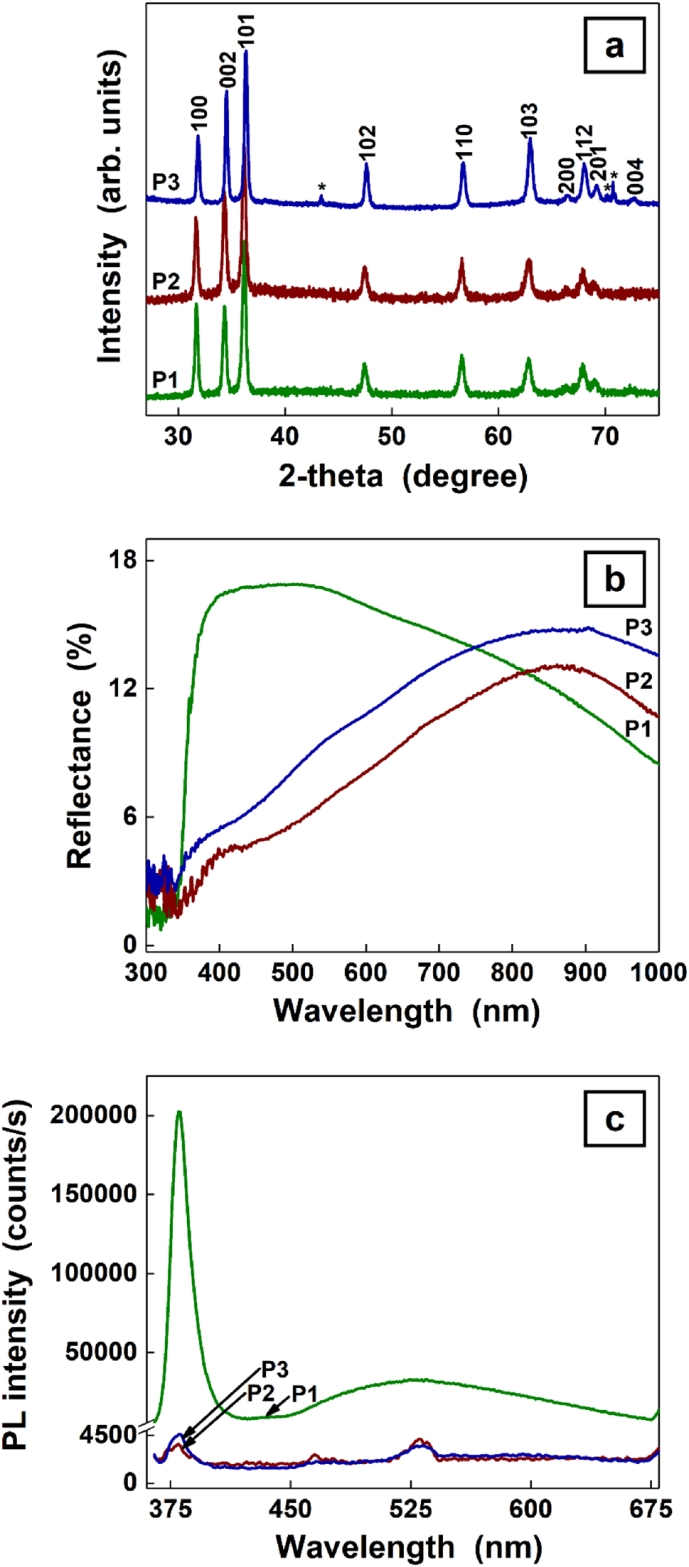
XRD patterns (**a**), reflectance spectra (**b**) and photoluminesce spectra (**c**) of ZnO nanowires (P1), ZnO–CuO core–shell nanowires (P2) and Ag nanoparticles-decorated ZnO–CuO core–shell nanowires (P3). *Peaks attributed only to zinc foil.

The structural and optical properties of the investigated samples were evaluated from data shown in Fig. 5. The XRD patterns of all samples are similar exhibiting the diffraction peaks indexed to the hexagonal wurtzite ZnO structure (ICDD 00-035-1451) and some diffraction peaks associated to the underlying Zn substrate. In the XRD patterns of the ZnO–CuO core–shell nanowires with or without Ag nanoparticles, the diffraction signature of the CuO and Ag had not been detected. In the reflectance spectra, two strong decreases can be observed one below ~ 400 nm for all samples containing nanowires and another below ~ 850 nm for ZnO–CuO core–shell nanowires with or without Ag nanoparticles due to the band-to-band transition in ZnO and CuO, respectively, the results being in agreement with those previously reported^5,6^. Additionally to these decrease peaks, the reflectance spectrum of Ag nanoparticles decorated ZnO–CuO core–shell nanowire sample discloses a shoulder of ~ 550 nm, which can be associated to the Ag plasmon band^54,55^. The presence, position and intensity of this band is strongly influenced by parameters of the Ag nanoparticles (size, shape, inter-particle distance, surface chemistry, surrounding environment, etc.)^56^. The photoluminescence spectrum of ZnO nanowires exhibits two emission bands: one intense and sharp in the UV domain centered at ~ 380 nm of excitonic origin^57^ and another one weak, broad covering almost all the visible range centered at ~ 530 nm originating from defect emission^57,58^. Although, the origin of this visible emission is still under debate, the generally accepted hypothesis considers the oxygen vacancies responsible for the appearance of this “green emission”^59,60^. It has to be noted that the presence of the oxygen vacancies can modify the wetting properties of the ZnO nanowires^21^. Moreover, a study focused on the origin of the “green emission” in ZnO nanostructures (including nanowires) prepared by a vapor phase transport method (also a dry preparation technique) emphasized that the oxygen gas concentration used during the ZnO growth influences the depth of the oxygen vacancies related defects^60^.

The photoluminescence spectra of ZnO–CuO core–shell nanowires with or without Ag nanoparticles show a decrease in the intensities of these two emission bands. The result can be explained considering that the CuO layer deposited on the surface of ZnO nanowires leads to a passivation effect reducing the ZnO surface oxygen related defects^61^ and to a separation effect of the charge carriers due to the formation of type-II band alignment between the two metal oxides^62,63^.

**Figure 6:**
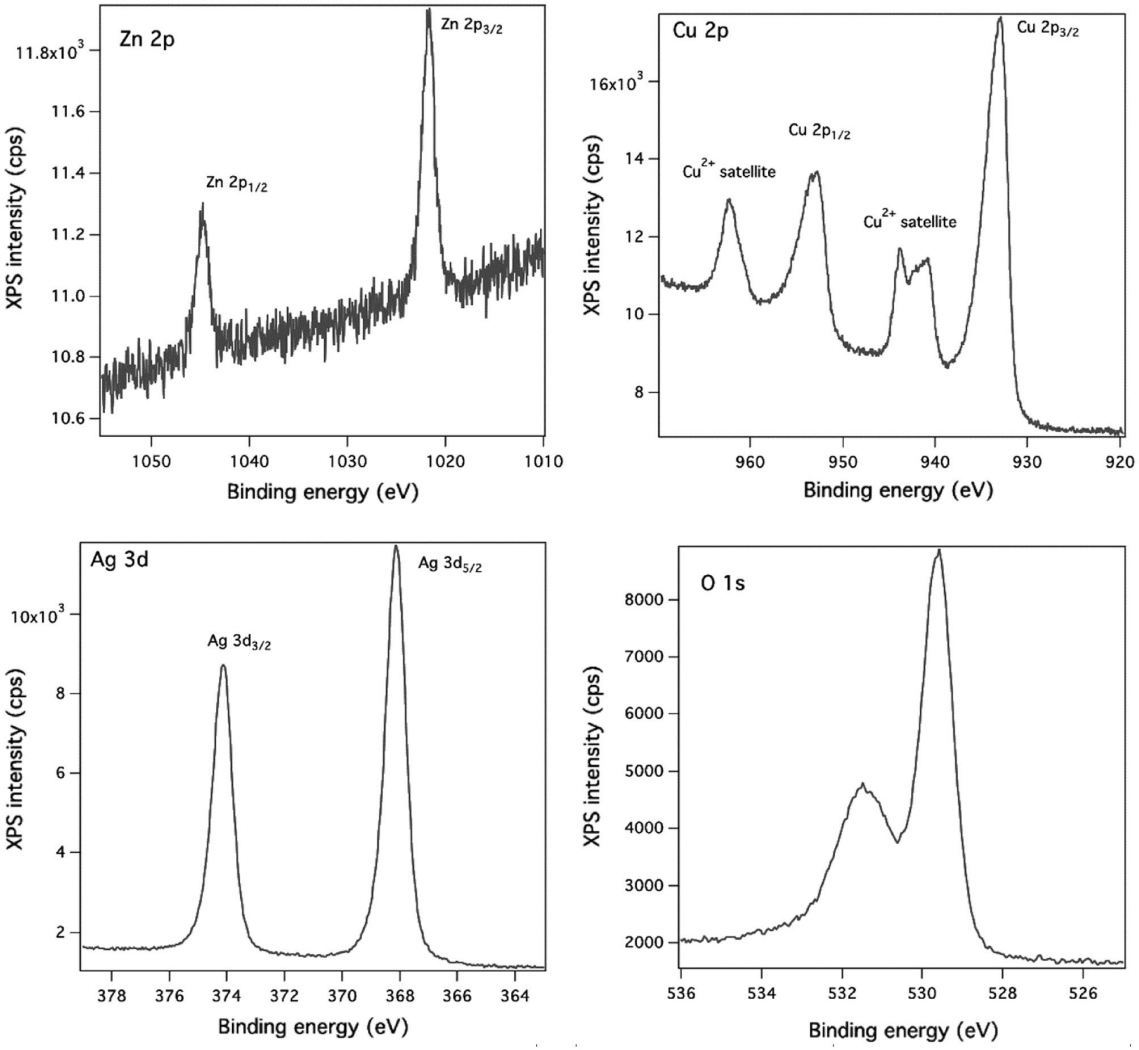
XPS spectra for the Zn 2p, Cu 2p, Ag 3d and O 1s core levels in Ag nanoparticles-decorated ZnO–CuO core–shell nanowires.

The Ag nanoparticles decorated ZnO–CuO core–shell nanowires sample was investigated by XPS in order to certify the chemical composition of the shell and nanoparticles and the oxidation states of each component, the XPS spectra being presented in Fig. 6. The peaks observed in the XPS spectra of Zn 2p, Cu 2p, Ag 3d and O 1s were indexed as follows: (i) at ~ 1021 eV and ~ 1045 eV to Zn 2p_3/2_ and Zn 2p_1/2_ levels of Zn^2+^ state in ZnO^64,65^, (ii) at ~ 933 eV and ~ 954 eV to Cu 2p_3/2_ and Cu 2p_1/2_ levels of Cu^2+^ state in CuO^64,65^, (iii) at ~ 368 eV and ~ 374 eV to Ag 3d_5/2_ and Ag 3d_3/2_ levels of Ag (0) state^66,67^, (iv) at ~ 529 eV to O^2−^ state in metal oxides^64,65^ and (v) at ~ 532 eV to C=O due to a contamination of the surfaces with carbonate from the environment. Further, the deconvolution obtained by Voigt profiles of the core level spectra of Zn 2p, Cu 2p, Ag 3d and O 1S (Fig. S2) were analyzed for evidencing the presence of other species of these elements, the following information being obtained: (i) Zn 2p_3/2_ core level—a narrow peak centered at 1021.7 eV due to Zn^2+^ state in ZnO^64,65^; (ii) Cu 2p_3/2_ core level—a broad peak with two components, one centered at 933.9 eV owed to Cu^2+^ state in CuO^64,65^ and other at 934.8 eV linked most probably to carbonate resulted from the slight contamination of the surface in the environment conditions and the satellite peaks at ~ 940–945 eV and ~ 964 eV characteristic only to the bivalent oxidation state of Cu, their presence proving the formation of CuO^64,65^; (iii) Ag 3d_5/2_ core level—a broad peak with two components, one centered at 368.1 eV associated to the free metallic silver atoms (Ag (0))^66,67^ and other centered at 368.7 eV related most probably to the positively charged silver atoms (Ag (0) + δ) resulted from the slight oxidation in air of the metallic atoms in the ambient conditions^68,69^ or from the silver interaction with a relatively high electronegative atom like oxygen)^70–72^. Consequently, the XPS results prove the presence of ZnO, CuO and Ag in the Ag nanoparticles decorated ZnO–CuO core–shell nanowires samples.

**Figure 7:**
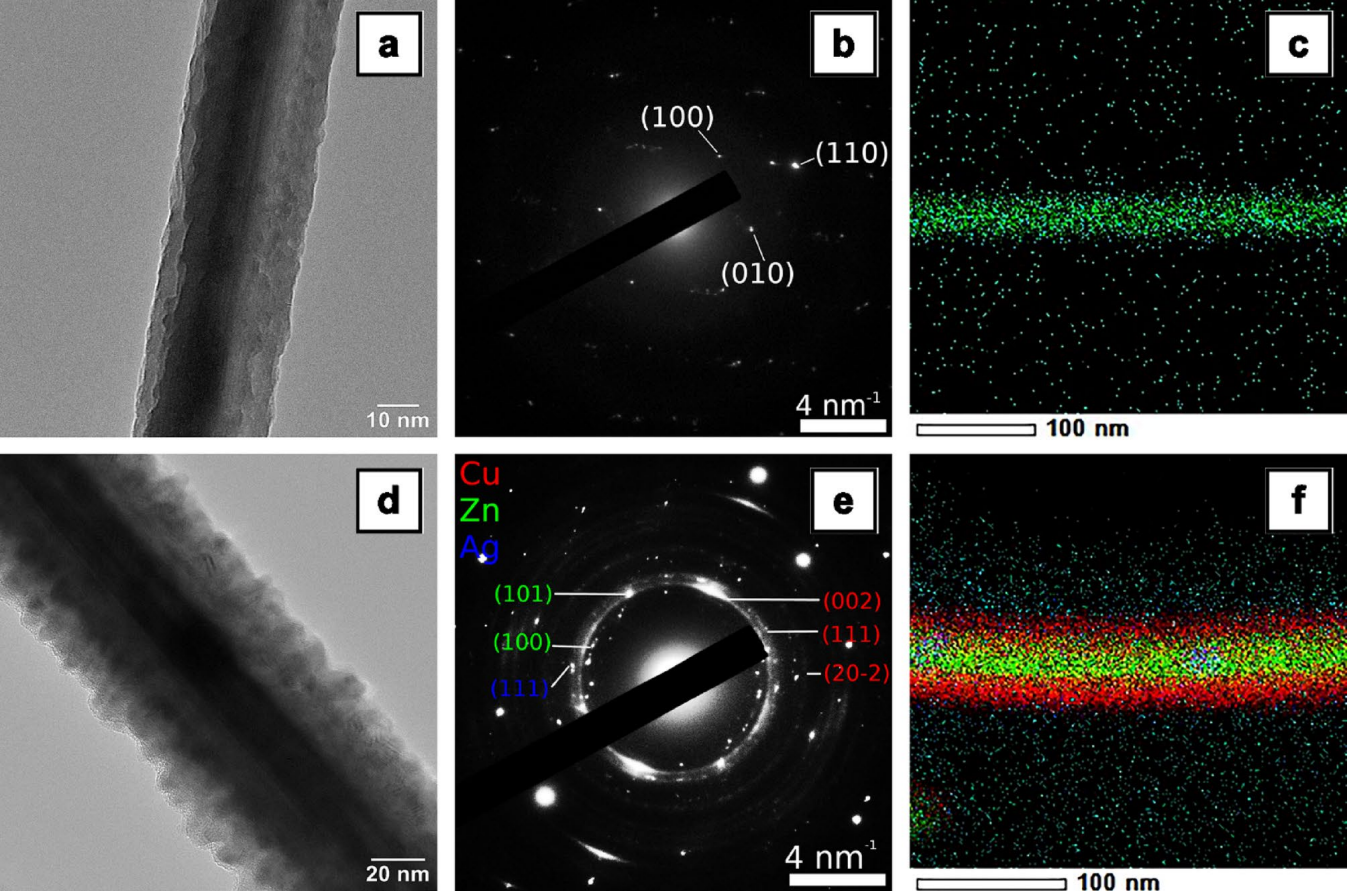
TEM images (**a**,**d**), SAED patterns (**b**,**e**) and EDX elemental mappings in STEM mode including the spatial distribution of the Zn, Cu and Ag elements (**c**,**f**) in the ZnO nanowires (**a**–**c**) and Ag nanoparticles-decorated ZnO–CuO core–shell nanowires (**d**–**f**).

Both ZnO nanowires and Ag nanoparticles decorated ZnO–CuO core–shell nanowires samples were investigated by TEM measurements in order to emphasize the core–shell morphology of the ZnO–CuO nanowires and their decoration with Ag nanoparticles, the data being shown in Fig. 7.

Hence, the TEM images of a single ZnO nanowire and a single Ag nanoparticles decorated ZnO–CuO core–shell nanowire evidence a cylindrical shape for both types of nanowires with diameter of ~ 30 nm and ~ 70 nm, respectively, in agreement with the FESEM images (Fig. 2). The SAED patterns certify a crystalline wurtzite structure for ZnO nanowire (in accordance with the XRD data from Fig. 5a) and three crystalline structures for Ag nanoparticles for Ag nanoparticles decorated ZnO–CuO core–shell nanowire: hexagonal wurtzite for the ZnO core, monoclinic for the CuO shell and face-centered cubic. The EDX elemental mapping of a single Ag nanoparticles decorated ZnO–CuO core–shell nanowire proves the formation of the core–shell morphology by the presence of Zn K in the inner part and Cu K in the outer part. In addition, the STEM image and EDX spectra in STEM mode acquired in two areas of a single Ag nanoparticles decorated ZnO–CuO core–shell nanowire (Fig. S3) emphasize the presence of both Ag nanoparticles distributed uniformly on the surface of the nanowire and Ag aggregated nanoparticles randomly positioned along the surface of the nanowire.

**Figure 8:**
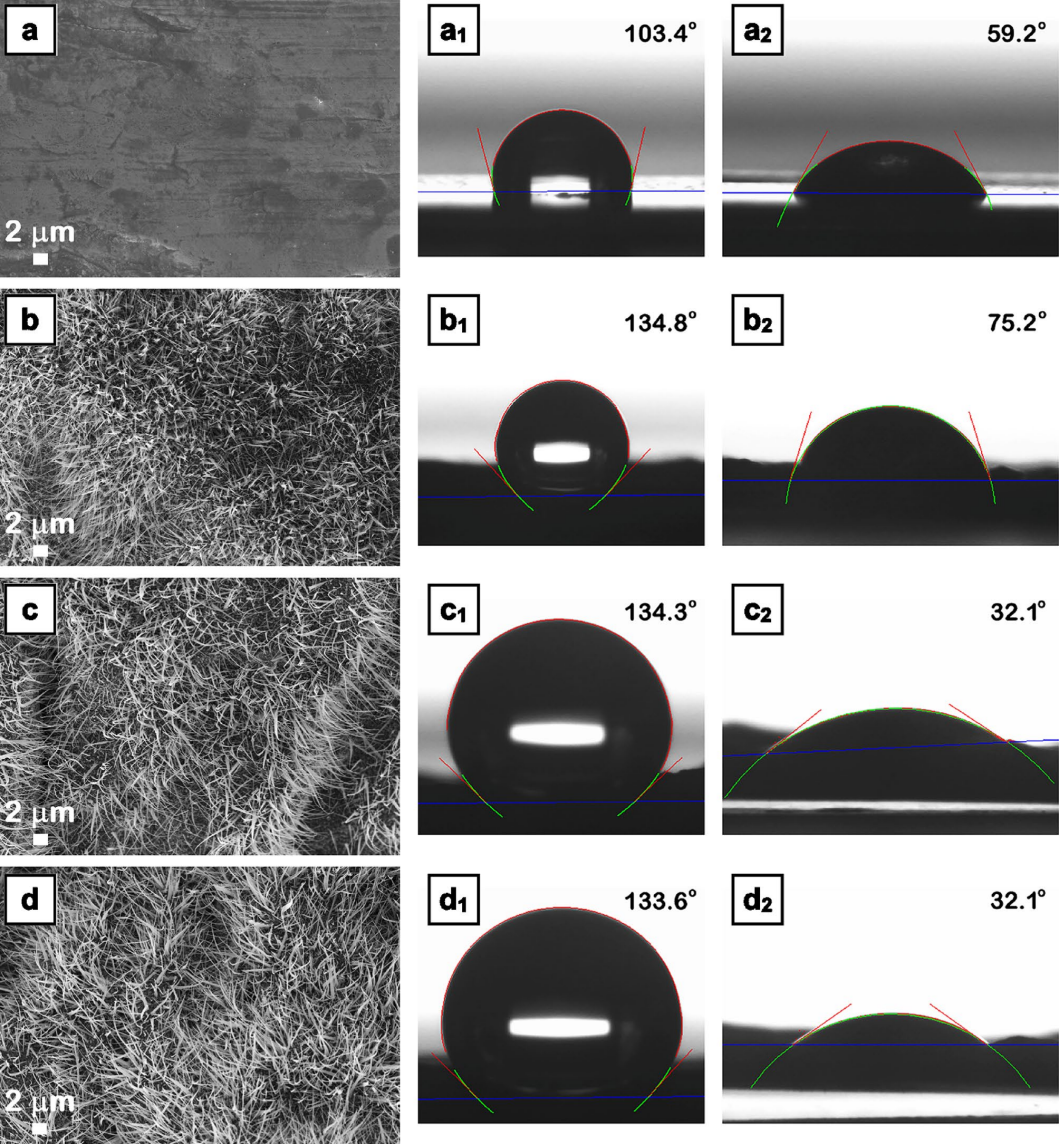
FESEM images (**a**–**d**) and optical photographs of the water droplets shape (**a**_**1**_–**d**_**1**_) and diiodomethane droplets shape (**a**_**2**_–**d**_**2**_) on the surface of Zn foil (**a**,**a**_**1**_,**a**_**2**_), ZnO nanowires (**b**,**b**_**1**_,**b**_**2**_), ZnO–CuO core–shell nanowires (**c**,**c**_**1**_,**c**_**2**_) and Ag nanoparticles-decorated ZnO–CuO core–shell nanowires (**d**,**d**_**1**_,**d**_**2**_). The contact angle is presented as the mean value.

Usually, the wettability of a solid surface is decided by the geometrical structure of the surface as well as its chemical composition. Hence, in the present case, the micro/nano-structuration of the metallic surface and the material-dependent surface free energy have a combined and significant effect on the surface wettability, this being a key parameter related to their potential applications in surfaces with antibacterial activity. Thus, the samples were investigated by CA and SFE measurements using water and diiodomethane as test liquids in order to evaluate their wetting properties, the obtained values being given in Fig. 8 and Table S1, respectively. The FESEM images from Figs. S1 and 8 confirm that, in all cases, the metallic foil is complete and uniformly covered with nanowires on large areas. Concerning the CA values, for water, the WCA value increases from 103° (P0) to 134° (P1, P2 and P3 samples) while for diiodomethane, the DIMCA increases from 59° (P0) to 75° (P1) and then decreases to 32° (P2 and P3). Consequently, regardless their chemical composition, all samples containing nanowires on their surfaces reveal a hydrophobic behavior.

It has to be noted that in the case of ZnO nanowires, the hydrophobic property is in accordance with that reported in the literature^19,20^ and even if the oxygen vacancy defects can modify their wetting properties^21^, it can be assume that the nanowire shape of the structures presented on the surface of the zinc foils plays the major role in their hydrophobicity taking into account that all samples containing nanowires have WCA of ~ 134°. Regarding the SFE components, the dispersive component is higher than the polar one for all samples. Thus, the lower polar component values (P1, P2 and P3 samples) leads to high WCA value for water droplets while the higher dispersive component (P2 and P3 samples) results in small DIMCA value for diiodomethane droplets.

**Figure 9:**
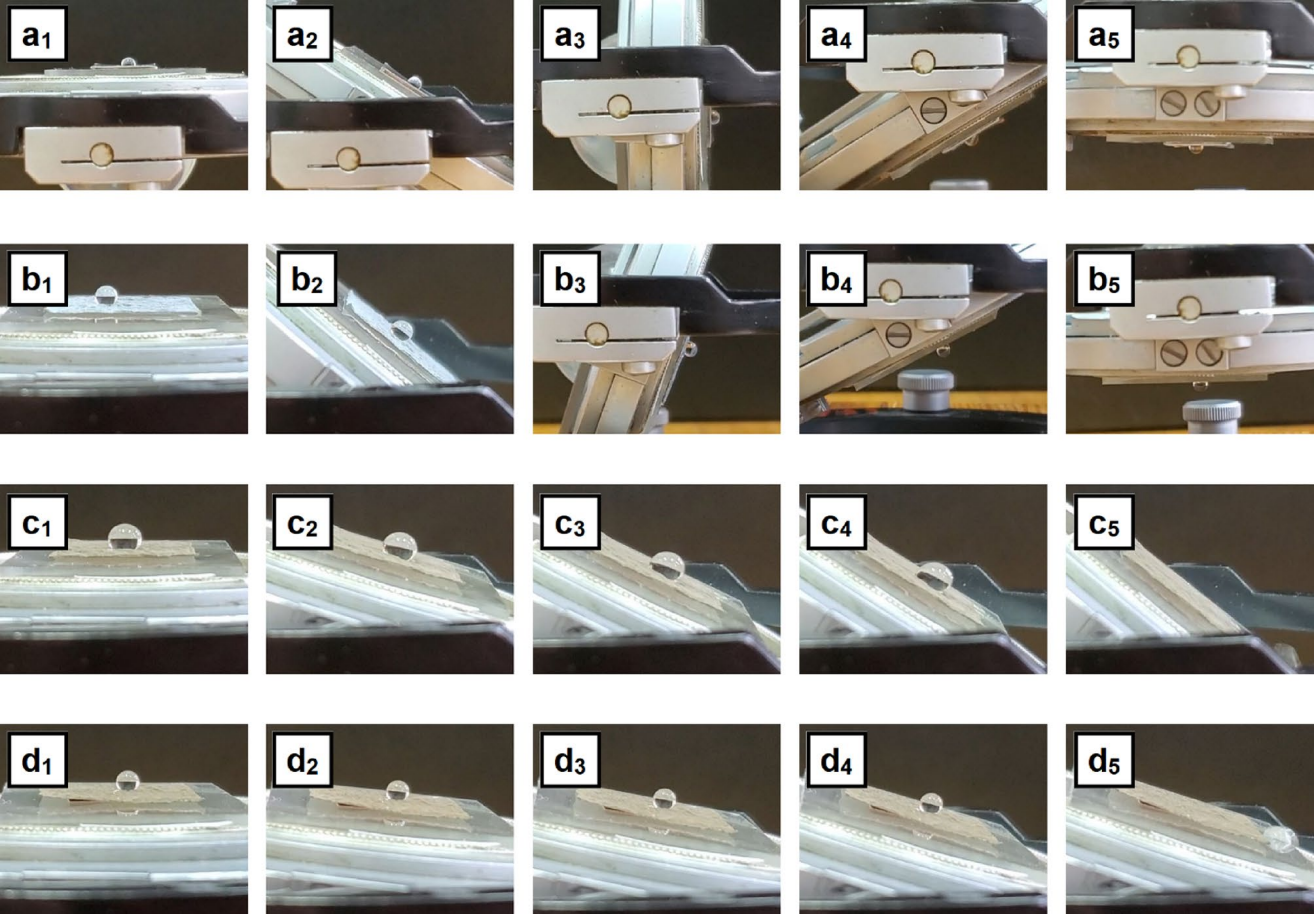
Sequences of snapshots taken from a video camera showing the high or low adhesion of water droplets on the surface of Zn foil (**a**_**1**_–**a**_**5**_), ZnO nanowires (**b**_**1**_–**b**_**5**_), ZnO–CuO core–shell nanowires (**c**_**1**_–**c**_**5**_) and Ag nanoparticles-decorated ZnO–CuO core–shell nanowires (**d**_**1**_–**d**_**5**_).

Further, the adhesion of the water droplets to the surface of the investigated samples were assessed, the images being given in Fig. 9. Also, in the case of water droplets, the *W*_*ad*_ values were estimated being presented in Table S1. It is known that when the drop slides on a sloping surface, the gravitational force can be considered a critical force to guide the movement of the drop, the energy required to roll/move the drop over a certain distance depending on the microscopic *W*_*ad*_. Hence, the *W*_*ad*_ can be a measure of the contact strength between two adjacent phases, being defined as the work required to separate these two phases (ex. liquid–solid phase boundary). Thus, the micro/nanostructured effect of the metallic surfaces consisting in the presence of the nanowire structures results in a lower *W*_*ad*_ value (~ 22 mN/m) in comparison to the smooth native metallic surface (~ 55 mN/m). It can be seen that the water droplet is highly adherent to the surface of the foils containing ZnO nanowires (similar to the water droplet behavior on the surface of Zn foil), resting stick, firmly pinned on the surface even when these were turned upside down. Instead, in the case of the foils having the surfaces covered with ZnO–CuO core–shell nanowires or Ag nanoparticles decorated ZnO–CuO core–shell nanowires, the water droplets rolled off very easily at slight tilt, the roll-off angle being evaluated at 55° for P2 or 25° for P3. The result can be explained taking into account the models concerning the three main states of a liquid droplet penetration in the textured (micro/nanostructured) surface: Wenzel, Cassie-Baxter and an intermediate state between them^73^.

Hence, the wetting regime for P1 sample can involve a stronger adhesion of the water droplet to the textured surface—the droplet is pinned to the surface as the air pockets are penetrated by the water while the wetting regime for P2 and P3 samples can presume a lower adhesion of the water droplet to the textured surface—the droplet sits on the top of the surface due to the air trapped underneath, in the gap between the nanostructures, preventing the penetration of the water. Such surfaces with hydrophobic behavior and low water droplet adhesion can be effective for antibacterial uses.

**Figure 10:**
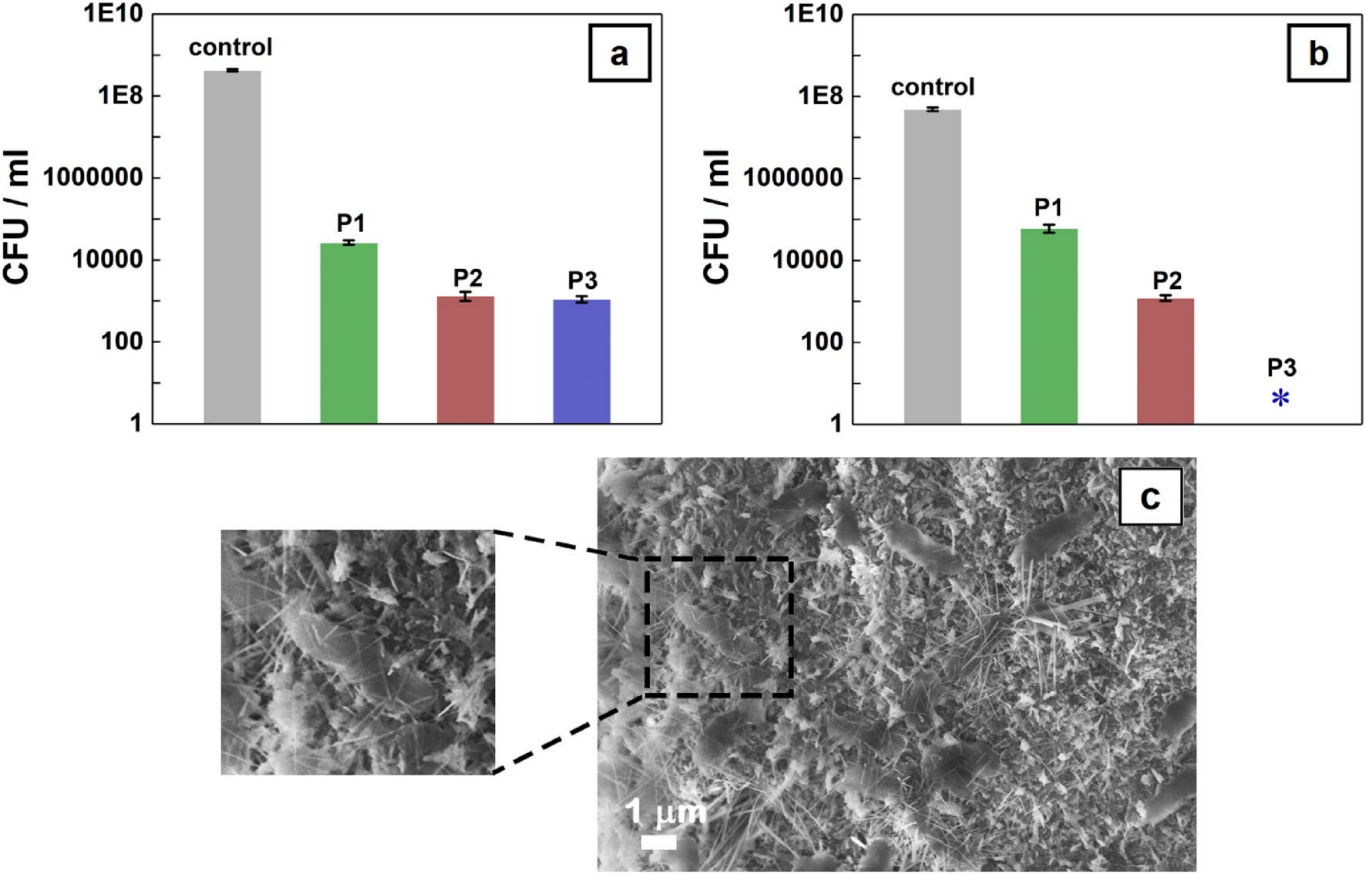
*E. coli* planktonic cells (**a**) and *S. aureus* planktonic cells (**b**) inhibition growth analysis of the ZnO nanowires (P1), ZnO–CuO core–shell nanowires (P2) and Ag nanoparticles-decorated ZnO–CuO core–shell nanowires (P3) and FESEM image of *E. coli* bacteria mechanically damaged by the nanowires.

In the following, the antibacterial activity of the nanostructured surfaces based on nanowire arrays was investigated against *E. coli* and *S. aureus*, the data being shown in Fig. 10a,b. The reduction in the bacterial growth is ~ 99.99% regardless both bacteria and nanowire composition types. Furthermore, no bacteria was found in the tests carried on *S. aureus* and Ag nanoparticles decorated ZnO–CuO core–shell nanowire arrays. Hence, it can be assume that the nanowire shape plays the significant role in achieving the excellent antibacterial response, the needle tip of the nanowire structures mechanically damaging the bacteria cells like a thorn that penetrates the membrane as can be seen in the FESEM image from Fig. 10c and in its detail. In addition, the hydrophobic and low water adhesion properties (in the case of P2 and P3 samples) can positively influence the antibacterial effect by limiting the contact area between the microbial suspension and the nanostructured surface. Still, various mechanisms can also assist the bacteria death. In our previous paper focused on the fabrication of the water stable photocatalysts based on ZnO–Cu_x_O core–shell nanowires^16^, we evidenced that a ZnO dissolution can occurs in the aqueous solutions and the presence of a CuO layer with an adequate thickness on the surface of the ZnO nanowires protects them against the dissolution in the aqueous media. Accordingly, the zinc ions (P1 sample) or the silver ions (P3 sample) generated in the aqueous media can adhere to the cell wall, increasing its permeability and further leading to the cell membrane rupture^74^ while the formation of ZnO–CuO p-n junction^16^ (P2 sample) and Ag-CuO Schottky barrier^75^ (P3 sample) can favor the separation of charge carrier pair, which further can improve the generation of highly ROS involved in the mechanism of bacteria killing. Consequently, the antibacterial activity of the samples due to the mechanically damaged bacteria membrane can be assist by a chemical contact killing bacteria mechanism.

The outcome of the experiments proves that such functional nanostructured surfaces based on silver nanoparticles decorated ZnO–CuO core–shell nanowire arrays with low water droplet adhesion and excellent bactericide effect can open new insights in the field of water repellent and antibacterial coatings.

## Conclusions

High-aspect-ratio silver nanoparticles decorated ZnO–CuO core–shell nanowire arrays were fabricated by combining low-cost and reproducible dry preparation techniques that can be relative easily scaled to large area. Hence, on zinc foils, ZnO nanowire arrays were grown by thermal oxidation in air, subsequently coated with a CuO layer by RF magnetron sputtering, the obtained ZnO–CuO core–shell nanowires being further decorated with Ag nanoparticles by thermal vacuum evaporation. The wettability measurements show that all investigated samples reveal hydrophobic behavior. Moreover, the native Zn foil and the ZnO nanowires arrays are featured by a high water droplet adhesion while ZnO–CuO core–shell nanowires arrays with or without Ag nanoparticles by a low water droplet adhesion. The antibacterial tests carried on *E. coli* and *S. aureus* emphasize that all nanostructured surfaces based on nanowire arrays present excellent antibacterial activity against both type of bacteria, the hydrophobic and low water adhesion behavior of the nanowire samples enhancing the antibacterial effect by limiting the contact area between the microbial suspension and the nanostructured surface. The nanowire shape plays the major role in achieving the excellent antibacterial response, the nanowire structures mechanically damaging the bacteria cells, process that can be assists by other mechanisms involving release of metal ions or formation of different type of junctions. This study emphasized that such functional surfaces can be viable candidates for water repellent surfaces with enhanced antibacterial activity.

## Supplementary Information

Supplementary Information.

## Data Availability

The datasets supporting the conclusions of the current study are presented in the manuscript and supporting information.
