# Supplementary material for: Silver nanoparticles decorated ZnO–CuO core–shell nanowire arrays with low water adhesion and high antibacterial activity
Source: Sci Rep. 2023 Jul 3;13:10698. doi: 10.1038/s41598-023-37953-w (PMC10318101; doi:10.1038/s41598-023-37953-w)
Supplement: Supplementary file 1 — Supplementary Information. [file 41598_2023_37953_MOESM1_ESM.docx]

Silver nanoparticles decorated ZnO-CuO core-shell nanowire arrays with low water adhesion and high antibacterial activity

Andreea Costas^1¥^, Nicoleta Preda^1¥*^, Irina Zgura^1^, Andrei Kuncser^1^, Nicoleta Apostol^1^, Carmen Curutiu^2^, Ionut Enculescu^1^

^1^National Institute of Materials Physics, Atomistilor 405A, 077125, Magurele, Romania

^2^University of Bucharest, Faculty of Biology, Microbiology Immunology Department, Aleea Portocalelor 1-3, 060101, Bucharest, Romania

^¥^ Nicoleta Preda and Andreea Costas contributed equally to this work.

^*^Corresponding author: Nicoleta Preda

Tel (office): + 40 21 3690185

Fax (office): + 40 21 3690177

e-mail: [nicol@infim.ro](mailto:nicol@infim.ro)


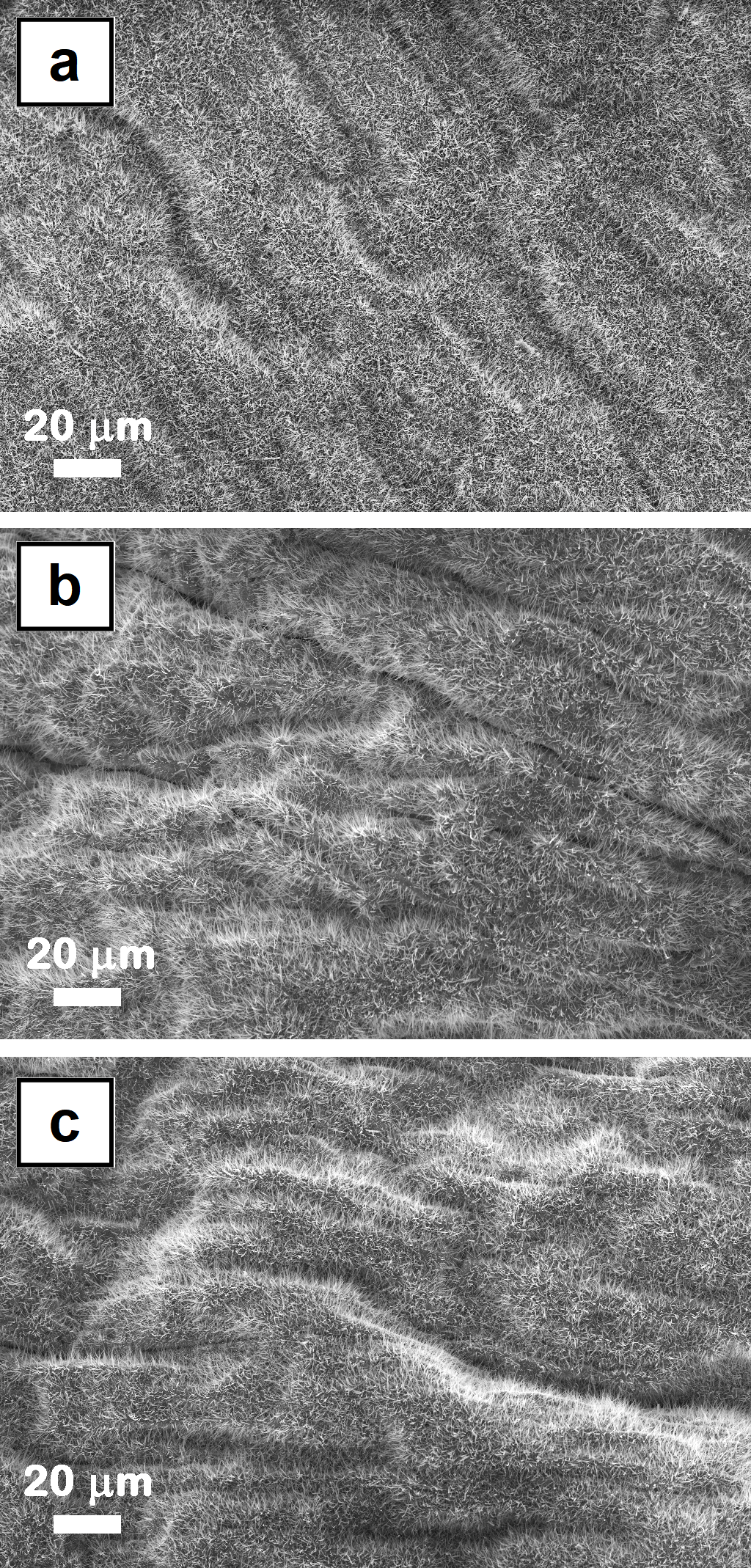


Figure S1. FESEM images at lower magnification of ZnO nanowires (a), ZnO-CuO core-shell nanowires (b) and Ag nanoparticles-decorated ZnO-CuO core-shell nanowires (c).


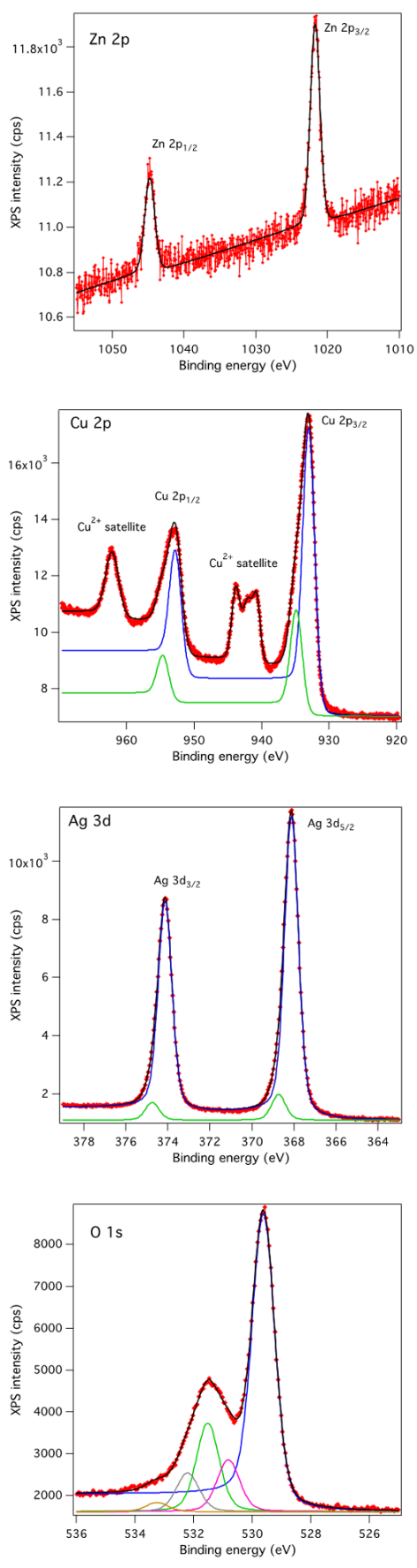


Figure S2. High resolution XPS spectra of the core level Zn 2p, Cu 2p, Ag 3d and O 1s in

Ag nanoparticles-decorated ZnO-CuO core-shell nanowires.


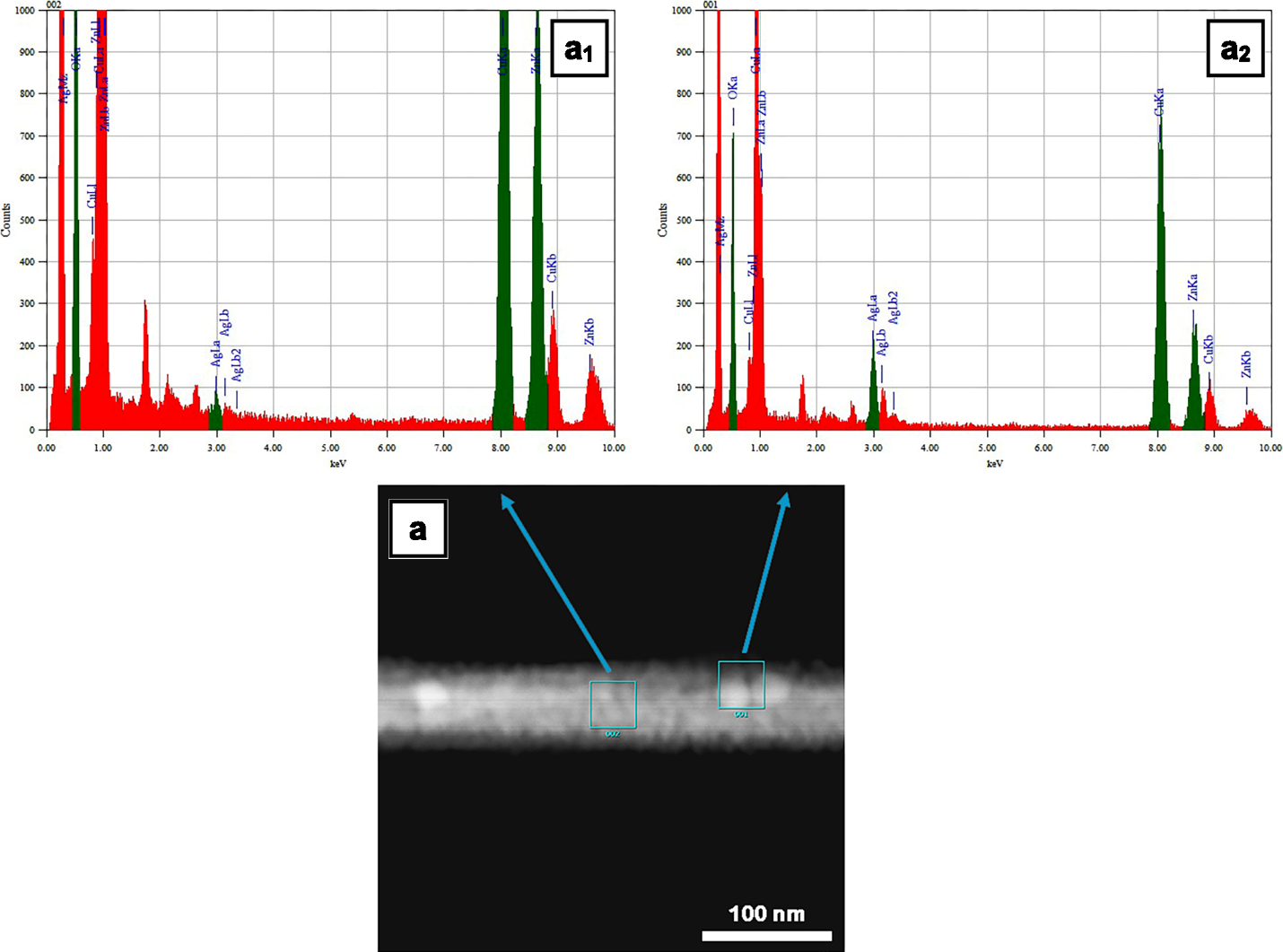


Figure S3. STEM image (a) and EDX spectra in STEM mode (a_1_, a_2_) acquired in two areas of a single Ag nanoparticles-decorated ZnO-CuO core-shell nanowire.

| **Sample** | ***γ_S_^p^* (mN/m)** | ***γ_S_^d^* (mN/m)** | ***γ_S_* (mN/m)** | ***W_ad_* (mN/m)** |
| --- | --- | --- | --- | --- |
| P_0_ | 0.1 | 29.6 | 29.7 | 55.99 |
| P_1_ | 3.0 | 24.6 | 27.6 | 21.55 |
| P_2_ | 11.2 | 55.7 | 66.9 | 21.95 |
| P_3_ | 10.8 | 55.5 | 66.4 | 22.64 |

Table 1. Surface energy (with its polar and dispersive components) and adhesion work of the investigated samples P_0_-P_3_ (the values were calculated taking into account the CA mean value obtained in the case of water droplet).
